# A Toll-Like Receptor 2 Pathway Regulates the *Ppargc1a/b* Metabolic Co-Activators in Mice with *Staphylococcal aureus* Sepsis

**DOI:** 10.1371/journal.pone.0025249

**Published:** 2011-09-26

**Authors:** Timothy E. Sweeney, Hagir B. Suliman, John W. Hollingsworth, Karen E. Welty-Wolf, Claude A. Piantadosi

**Affiliations:** 1 Department of Pathology, Duke University Medical Center, Durham, North Carolina, United States of America; 2 Department of Anesthesiology, Duke University Medical Center, Durham, North Carolina, United States of America; 3 Department of Immunology, Duke University Medical Center, Durham, North Carolina, United States of America; 4 Department of Medicine, Duke University Medical Center, Durham, North Carolina, United States of America; McGill University, Canada

## Abstract

Activation of the host antibacterial defenses by the toll-like receptors (TLR) also selectively activates energy-sensing and metabolic pathways, but the mechanisms are poorly understood. This includes the metabolic and mitochondrial biogenesis master co-activators, *Ppargc1a* (PGC-1α) and *Ppargc1b* (PGC-1β) in *Staphylococcus aureus* (*S. aureus*) sepsis. The expression of these genes in the liver is markedly attenuated inTLR2^−/−^ mice and markedly accentuated in TLR4^−/−^ mice compared with wild type (WT) mice. We sought to explain this difference by using specific TLR-pathway knockout mice to test the hypothesis that these co-activator genes are directly regulated through TLR2 signaling. By comparing their responses to *S. aureus* with WT mice, we found that MyD88-deficient and MAL-deficient mice expressed hepatic *Ppargc1a* and *Ppargc1b* normally, but that neither gene was activated in TRAM-deficient mice. *Ppargc1a/b* activation did not require NF-kβ, but did require an interferon response factor (IRF), because neither gene was activated in IRF-3/7 double-knockout mice in sepsis, but both were activated normally in Unc93b1-deficient (3d) mice. Nuclear IRF-7 levels in TLR2^−/−^ and TLR4^−/−^ mice decreased and increased respectively post-inoculation and IRF-7 DNA-binding at the *Ppargc1a* promoter was demonstrated by chromatin immunoprecipitation. Also, a TLR2-TLR4-TRAM native hepatic protein complex was detected by immunoprecipitation within 6 h of *S. aureus* inoculation that could support MyD88-independent signaling to *Ppargc1a/b*. Overall, these findings disclose a novel MyD88-independent pathway in *S. aureus* sepsis that links TLR2 and TLR4 signaling in innate immunity to *Ppargc1a/b* gene regulation in a critical metabolic organ, the liver, by means of TRAM, TRIF, and IRF-7.

## Introduction

TLR cell surface receptors that activate innate immunity form specific dimers in response to conserved pathogen-associated molecular patterns (PAMPs) [1]. In particular, TLR1-2 and TLR2-6 heterodimers bind bacterial Gram-positive lipopeptides, while TLR4 homodimers bind Gram-negative lipopolysaccharide (LPS) [1]. All known TLRs, except TLR3, signal through the MyD88 adaptor, and canonical TLR2 and TLR4 pathways operate through MyD88 and MAL (TIRAP) to trigger pro-inflammatory gene activation through NF-κB and mitogen-activated protein kinases [2], [3]. TLR2^−/−^ cells show attenuated cytokine responses to Gram-positive pathogens, such as *S. aureus*, while MyD88^−/−^ macrophages show no NF-κB-mediated TNF-α and IL-6 production [4]. TLR3 activates TRIF (TICAM-1) and TRAM (TICAM-2) to activate interferon response factors-3 and -7 (IRF-3 and IRF-7) [5], [6], [7]. Some of these adaptor functions overlap, and TLR2 and TLR4 may also signal non-canonically through TRIF [8]. For instance, TLR2 responds to viral ligands through TRIF to activate IRF-3/7 in a MyD88-independent manner [9]. Also, TLR4 is activated by pathogenic *S. aureus* and Gram-positive cell wall components [10], [11], [12], [13], [14], [15], [16].

Immune hyper-activation in sepsis produces metabolic stress, e.g. from cytokine synthesis, fever, catecholamine release, NO production, and changes in carbon substrate and oxygen utilization [17]. In this setting, several energy-producing metabolic and catabolic pathways are activated in response to the increased cellular ATP and substrate requirements, but this also generates, excessive reactive oxygen and nitrogen species, and this set of conditions may promote mitochondrial damage and metabolic dysregulation [18], [19], [20]. The energy-protective responses of the cell also include mitochondrial biogenesis, which is initiated through nuclear gene activation [21], [22] controlled by “master” co-activator genes, e.g. the peroxisome proliferator-activated receptor gamma co-activators, *Ppargc1a*, *Ppargc1b*, and *Pprc* [23], [24], [25], whose protein products (PGC-1α PGC-1β and PRC) partner with transcription factors that regulate and enhance mitochondrial quality control [26]. PGC-1 is also critically involved in lipid homeostasis and glucose metabolism [27], [28], especially in the liver, wherein heterozygosity of PGC-1α reduces the level of gene expression, leading to impaired fatty acid oxidation, steatosis, and insulin resistance [28]— the metabolic hallmarks of sepsis.

Under the metabolic stress of *S. aureus* sepsis, *Ppargc1a* and *Ppargc1b* are up-regulated synchronously, but independently of *Pprc*. In peritonitis, *Ppargc1a*/*Ppargc1b* mRNA levels increase ∼5-fold in the liver in WT mice, but neither mRNA increases in TLR2^−/−^ mice, and both increase by 10–15-fold in TLR4^−/−^ mice, in part through suppression of microRNA-mediated mRNA degradation [29]. Of further interest, both *Ppargc1* genes are up-regulated in sepsis through an unknown cascade involving the TLR2 and TLR4 signaling pathways. These findings led us to postulate that *S. aureus* infected mice up-regulate *Ppargc1a*/*Ppargc1b* through a unique arrangement of TLR2/TLR4 and adaptor proteins that links innate immunity to cell metabolism and mitochondrial biogenesis in the liver, a crucial metabolic and immune organ.

Our findings indicate that hepatic *Ppargc1a*/*Ppargc1b* up-regulation in *S. aureus* sepsis is independent of MyD88 and MAL and does not require NF-κB, but relies instead on a novel TLR2 pathway involving TRAM, TRIF, and IRF-3/7. Studies of *Ppargc1* regulation in Unc93b1^−/−^ (3d) mice also indicate a lack of involvement of nucleic acid sensing TLRs (TLR3, 7–9), and we identify a post-inoculation interaction of TRAM with TLR2 and TLR4 that may represent a platform for TLR2 signaling to TRAM and IRF-3/7.

## Results

### Murine Model

*S. aureus* sepsis in mice produced by fibrin-clot implantation is characterized by hepatic TLR2 and TLR4 up-regulation without involvement of exogenous LPS [22], [29]. The liver also demonstrates an early up-regulation of the PGC-1 co-activator family of genes, but *Ppargc1a* and *Ppargc1b* are not up-regulated in TLR2^−/−^ mice and are amplified in TLR4^−/−^ mice [29].

### Liver cytokine expression in WT, TLR2^−/−^, and TLR4^−/−^ mice

**Figure 1:**
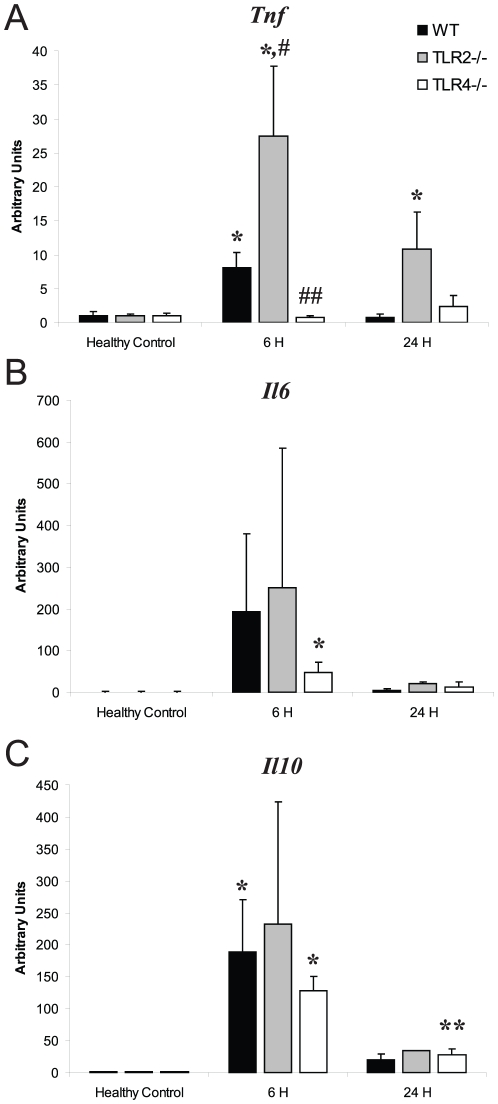
*Tnf*, *Il6*, and *Il10* mRNA expression. Hepatic mRNA levels of *Tnf* (A), *Il6* (B), and *Il10* (C) were measured in WT, TLR2^−/−^, and TLR4^−/−^ mice at 0 h (healthy control; HC), 6 h and 24 h PI in *S. aureus* sepsis. For each strain, n≥3 mice at each time point were compared with HC of the same strain. * *P*<0.05, ** *P*<0.01; # indicates higher and ## lower values than WT. Vertical bars are SD.

In order to check for appropriate cytokine responses to *S. aureus*, we measured *Tnf*, *Il6*, and *Il10* levels by Q-PCR in the liver in the peritonitis model (Fig. 1). All three cytokines were up-regulated in WT mice by 6 h PI, and declined towards baseline by 24 h. TLR2^−/−^ mice showed greater increases in all three cytokines than WT mice at 6 h PI, but statistically only *Tnf* levels were higher (WT *Tnf* 6 h PI: 8.04±2.32; TLR2^−/−^ *Tnf* 6 h PI: 27.51±10.29; *P*<0.05). In contrast, TLR4^−/−^ mice had depressed cytokine up-regulation compared with WT, but between the two strains only *Tnf* was statistically different at 6 h PI (TLR4^−/−^ *Tnf* 6 h PI: 0.69±0.28; *P*<0.01 vs. WT). Since *Tnf* production after *S. aureus* required TLR4, we checked LPS levels by the Limulus assay and detected only 0.04 ng LPS per clot. These abdominal clots undergo lysis over several days, so the mice absorbed less than 0.04 ng of exogenous LPS each day.

### NF-κB activation

**Figure 2:**
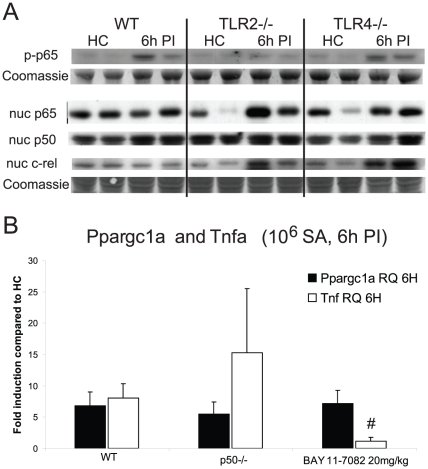
Nuclear p65, p50, and c-rel, and whole-cell phospho-p65. Immunoblots are shown for NF-κB family members in nuclear extracts and in whole-liver extracts from WT, TLR2^−/−^, and TLR4^−/−^ mice in HC and at 6 h PI (A). *Ppargc1a* and *Tnf* mRNA levels in *S. aureus* sepsis (B). *Ppargc1a* and *Tnf* mRNA levels at 6 h PI (compared to HC) were measured in WT, p50^−/−^, and BAY-11-7082-treated mice (n = 3 mice of each strain); * *P*<0.01 compared with WT *Tnf* levels at 6 h PI. Vertical bars are SD.

The unexpected increase in NF-κB-related cytokine production exhibited by TLR2^−/−^ mice in response to *S. aureus* was evaluated further in liver homogenates and nuclei from healthy control (HC), WT, TLR2^−/−^, and TLR4^−/−^ mice. We checked NF-κB activation by probing whole cell extracts for phospho-ser276-p65, and found p65 phosphorylation in WT and TLR4^−/−^ mice, but not in TLR2^−/−^ mice (Fig. 2A). Nuclear p65 protein in WT mice was comparable among HC mice and stable at 6 h PI, while HC TLR2^−/−^ and TLR4^−/−^ mice had variable nuclear p65 levels pre-infection (intra-experiment variability) and between-strain similarity in nuclear p65 levels at 6 h PI. Thus, the p65 phosphorylation and p65 nuclear patterns did not correspond. Nuclear p50 was detected in similar amounts in the HC mice of the three strains and did not increase 6 h PI. Nuclear cRel levels were stable at 6 h PI in WT mice, but increased in TLR4^−/−^ and TLR2^−/−^ mice. Thus, NF-κB activation in the liver after *S. aureus* inoculation was variable in the TLR-deficient strains and no pattern found that was consistent with *Pparg1a/b* mRNA expression.

The role of NF-κB on *Ppargc1a* activation was examined after *S. aureus* sepsis in two ways. WT mice were injected with an inhibitor of IkB-α phosphorylation, BAY-11-7082 [30] at 20 mg/kg [31], [32], and then inoculated with *S. aureus*. IkB-α binds preferentially to the p65 homodimer or to the p50–p65 heterodimer [33]; thus, BAY-treated mice showed no nuclear translocation of p50/p65. NF-κB activity in *S. aureus* sepsis was also evaluated in p50^−/−^ mice (the p65 knockout is lethal) by Q-PCR for *Tnf* mRNA compared with *Ppargc1a* mRNA. BAY-treated mice had no increase in *Tnf* expression at 6 h PI (WT: 8.0-fold PI vs. HC; BAY: 1.1-fold PI vs. HC; WT vs. BAY, *P*<0.01; Fig. 2B), thus *Tnf* induction depended on p50/p65 activation. The p50^−/−^ mice showed more variability in *Tnf* activity at 6 h PI (P = NS compared to WT), but *Tnf* mRNA was still induced. *Ppargc1a* mRNA was measured in BAY-treated WT and in p50^−/−^ mice, and neither experiment produced significantly different *Ppargc1a* mRNA levels compared with controls (P = NS at 6 h PI). Thus, *Ppargc1a* induction after *S. aureus* did not track TNF-α production and was independent of classical NF-κB activation.

### TLR-adaptor-deficient mice and *S. aureus* sepsis

**Figure 3:**
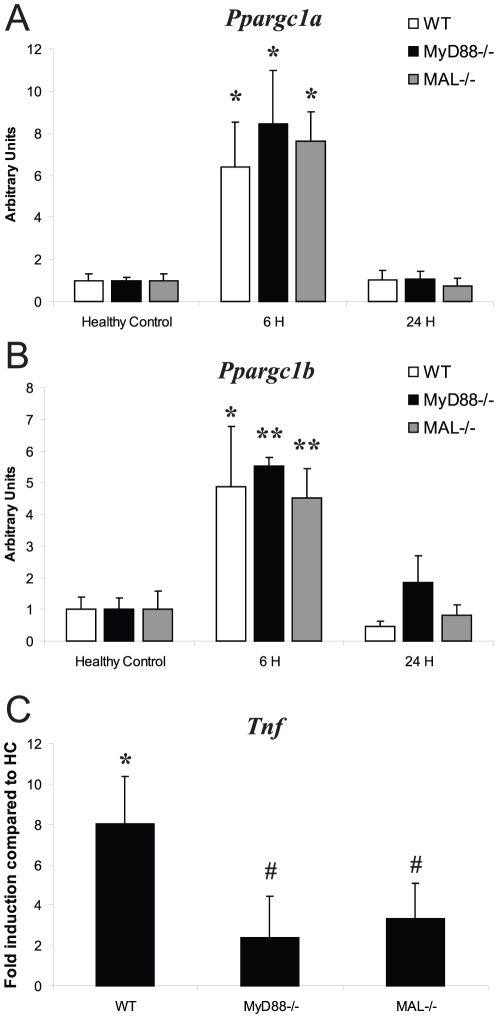
*Ppargc1a*, *Ppargc1b*, and *Tnf* mRNA levels in *S. aureus* sepsis. *Ppargc1a* (A) and *Ppargc1b* (B) mRNA levels were measured in WT, MyD88^−/−^, and MAL^−/−^ mice in healthy controls (HC) and at 6 h and 24 h PI by Q-PCR, together with *Tnf* mRNA levels (C) at 6 h PI (fold-induction compared to HCs; n≥3 mice at each point for each strain); * *P*<0.05, ** *P*<0.01 compared to HC of the same strain. Vertical bars are SD.

To explore the mechanism of *Ppargc1a/b* gene induction, MyD88^−/−^ and MAL^−/−^ mice were exposed to sepsis. MyD88^−/−^ mice do not activate NF-κB in response to *S. aureus* due to the lack of the TLR2 adapter molecule [4], [34], [35]. In MyD88^−/−^ and MAL^−/−^ mice, *Ppargc1a* and *Ppargc1b* mRNA induction relative to HC mice were the same as for WT mice. At 6 h PI in MyD88^−/−^ mice, *Ppargc1a* increased 8.5-fold vs. HC (*P*<0.05), and *Ppargc1b* increased 5.5-fold vs. HC (*P*<0.01). In MAL^−/−^ mice, *Ppargc1a* increased 7.6-fold vs. HC (*P*<0.01), and *Ppargc1b* increased 4.5-fold vs. HC (*P*<0.01) (Figs. 3A and 3B). However, neither strain showed *Tnf* up-regulation comparable to WT mice, which is consistent with impairment of NF-κB activation (Fig. 3C). We concluded that hepatic *Ppargc1a* and *Ppargc1b* are not under NF-κB control in *S. aureus* sepsis and are regulated in a MyD88- and MAL-independent manner.

**Figure 4:**
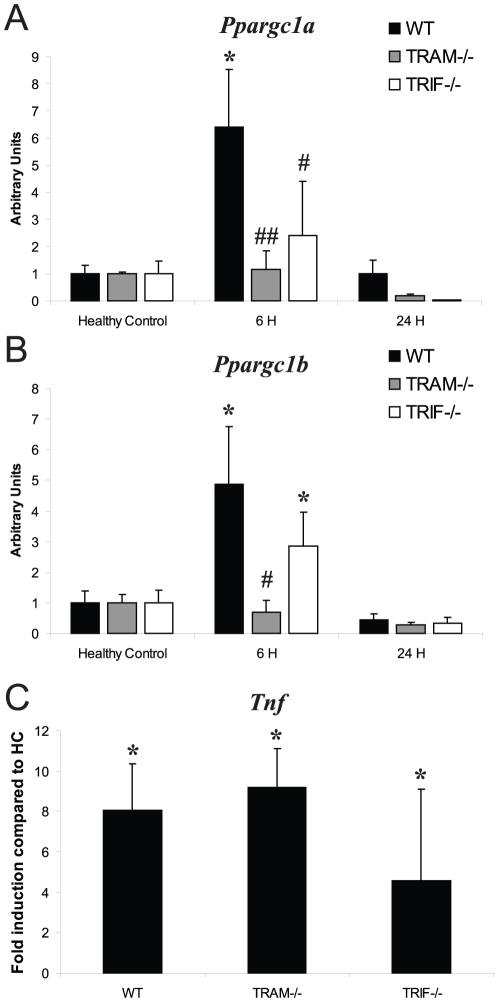
*Ppargc1a*, *Ppargc1b*, and *Tnf* mRNA levels in *S. aureus* sepsis. Hepatic mRNA levels of (A) *Ppargc1a* and (B) *Ppargc1b* were measured in WT, TRAM^−/−^, and TRIF^−/−^ mice in healthy controls (HC) and at 6 h and 24 h PI by Q-PCR, compared with mRNA levels of (C) *Tnf* at 6 h PI (fold-induction compared to HC; n≥3 mice at each point for each strain); * *P*<0.05 compared to HC of the same strain; #, *P*<0.05, ##, *P*<0.01 compared to WT data at 6 h. Bars are SD.

Since only four TLR adaptors are known, and the phenotype of MAL^−/−^ and MyD88^−/−^ mice did not match the TLR2^−/−^ mice, we considered the possibility that TLR2 could signal through TRAM and/or TRIF to induce *Ppargc1a/b* expression. We therefore exposed TRAM^−/−^ mice and TRIF^−/−^ mice to *S. aureus* and found that they did not up-regulate *Ppargc1a* at 6 h PI (TRAM^−/−^: 1.1-fold and TRIF^−/−^: 2.3-fold vs. HC, P = NS, and *P*<0.05 compared to WT at 6 h for both) (Fig. 4A). TRAM^−/−^ mice failed to up-regulate *Ppargc1b*, while TRIF^−/−^ mice showed some *Ppargc1b* activation at 6 h PI, but this was much less than for WT mice (TRAM^−/−^: 0.7-fold vs. HC, *P*<0.05 compared to WT at 6 h; TRIF^−/−^: 2.8-fold vs. HC, *P* = NS compared to WT at 6 h, *P*<0.05 compared to HC) (Fig. 4B). This indicated that the gene induction was dependent on TRAM and partly dependent on TRIF. Neither TRAM^−/−^ nor TRIF^−/−^ mice showed a significant difference in *Tnf* production compared with WT mice (Fig. 4C). Thus, TLR2 signaling for *Ppargc1a*/b gene induction operates through TRAM and TRIF because the absence of either interferes with the response.

### IRF-3 and IRF-7 activation and *Ppargc1a* transcription

**Figure 5:**
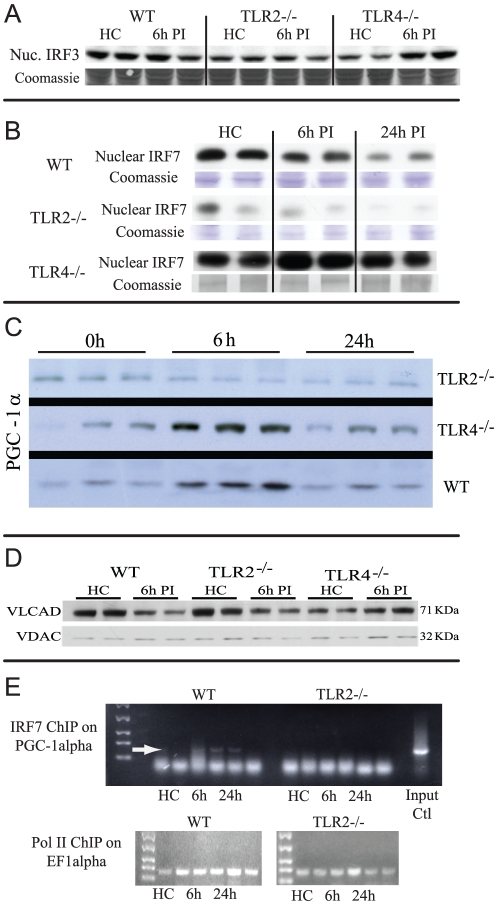
Nuclear immunoblots for IRF-3 (A) and IRF-7 (B). Immunoblots are shown for IRF-3 and IRF-7 in nuclear extracts from WT, TLR2^−/−^, and TLR4^−/−^ mice in HC and at 6 h PI (One of duplicate experiments with two mice per strain). (C) Immunoblots for PGC-1α protein in WT, TLR2^−/−^ and TLR4^−/−^ mice at 0, 6, and 24 h after *S aureus* inoculation. Equal protein loading was confirmed by Coomassie blue staining. (D) Immunoblots for the mitochondrial VLCAD fatty acid oxidation enzyme in HC and at 6 h PI in WT, TLR2^−/−^, and TLR4^−/−^ mice. Porin is a mitochondrial reference protein. (E) Chromatin Immunoprecipitation. ChIP for IRF7 binding on the *Ppargc1a* promoter at −289 bp from TSS. WT and TLR2^−/−^ mice (HC, 6 h PI, and 24 h PI) were tested. Arrow shows the position of the binding. Pol II pull-downs on EF1a are shown as loading controls.

The IRF-3 and IRF-7 transcription factors are the major known effectors of TRAM and TRIF activity, and these were assayed in WT and TLR2^−/−^ mice. IRF-3 and IRF-7 are constitutive and translocate to the nucleus upon activation [36], [37]; however, immunoblots did not suggest differences in nuclear IRF-3 protein levels between HC mice and WT and TLR2^−/−^ mice, but there was a small increase in TLR4^−/−^ mice at 6 h PI (Fig. 5A). In WT mice, nuclear IRF-7 showed little change at 6 h PI followed by a decline at 24 h PI, but TLR2^−/−^ mice showed a markedly low baseline level of nuclear IRF-7 and a further decrease at 6 h, whereas TLR4^−/−^ mice showed a marked increase in nuclear IRF-7 at 6 h post-inoculation (Fig. 5B). Thus, nuclear IRF-7 levels and nuclear IRF-7 translocation were deficient in TLR2^−/−^ mice and fit the pattern of *Ppargc1a* and *Ppargc1b* mRNA expression in TLR2^−/−^ [38] and in TRAM^−/−^ and TRIF^−/−^ mice.

The translation of *Ppargc1a* mRNA was checked by comparing the expression levels of total PGC-1α protein in WT, TLR2^−/−^, and TLR4^−/−^ mice after *S. aureus* inoculation (Fig. 5C). PGC-1α was up-regulated in WT and TLR4^−/−^ mice, but not inTLR2^−/−^ mice. We also monitored mitochondrial levels of the fatty acid oxidation enzyme, very long-chain specific acyl-CoA dehydrogenase (VLCAD), which is strongly regulated by PGC-1α. Hepatic VLCAD levels decreased in WT and especially in TLR2^−/−^ mice in sepsis, but increased in TLR4^−/−^ mice relative to the outer membrane reference protein porin (Fig. 5D).

The *Ppargc1a* and *Ppargc1b* promoter regions were examined for interferon-sensitive response elements (GAAANNGAAANN) where IRF-3 and IRF-7 binding occurs [39] and sites were found in both with close homology to the IRF-7 consensus. One *Ppargc1a* site around −289 Bp from the transcription start site (TSS) had a conserved ISRE in mouse and human genes (Table S1). For this site, we performed chromatin immunoprecipitation assays for IRF-7 and found that it was active in WT mice, but not in TLR2^−/−^ mice (Fig. 5E). Positive (RNA Polymerase II and transcription factor EF1α) and negative (negative IgG) controls confirmed specificity for IRF-7 occupancy of the *Ppargc1a* promoter.

**Figure 6:**
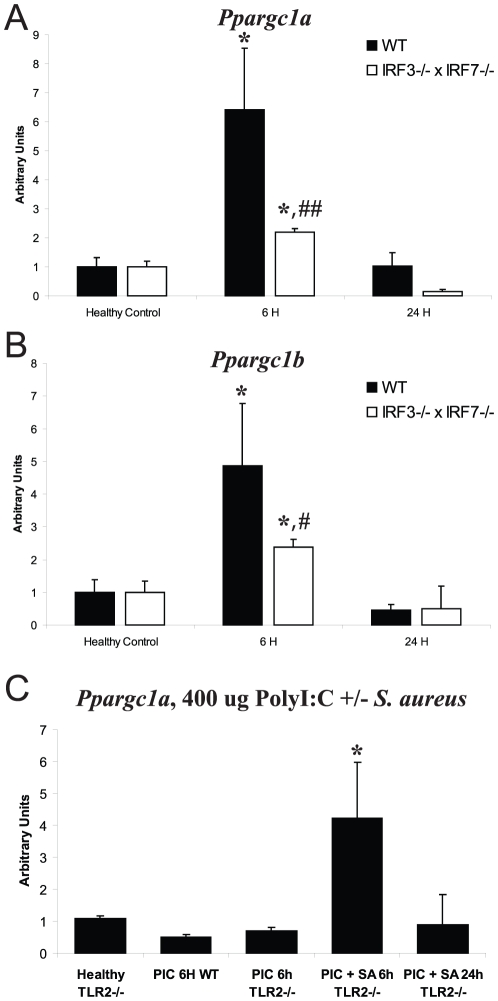
*Ppargc1a* and *Ppargc1b* mRNA levels in *S. aureus* sepsis. Hepatic levels of (A) *Ppargc1a* and (B) *Ppargc1b* mRNA were measured in WT and IRF3/7^−/−^ mice in healthy controls (HC) and at 6 h and 24 h PI (n≥3 at each point for each strain). (C) *Ppargc1a* mRNA levels after PolyI:C treatment with or without *S. aureus* sepsis. *Ppargc1a* mRNA levels were measured in WT and TLR2^−/−^ mice in healthy controls (HC), in mice dosed with 400 ug PolyI:C, and in mice given PolyI:C plus *S. aureus* sepsis at 6 h and 24 h PI (n = 3 mice at each point for each strain *, *P*<0.05 compared to HC of the same strain; ##, *P*<0.05, #, *P* = 0.08 compared to WT at 6 h). Vertical bars are SD.

IRF-3^−/−^×IRF-7^−/−^ mice on a C57bl/6J background (IRF-3/7 DKO) [40] were also tested in the *S. aureus* model. IRF-3/7 DKO mice did not induce *Ppargc1a* or *Ppargc1b* in the liver to nearly the extent of WT mice (Figs. 6A and 6B). These data further support TLR2 activation of a TRAM/TRIF→IRF-3/7→*Ppargc1a/b* response to *S. aureus*.

We performed functional rescue experiments of the TLR2^−/−^ *Ppargc1a*/b phenotype in the *S. aureus* model. Since TLR3 activates IRF-3 and IRF-7, we used polyinosinic-polycytidylic acid (polyI:C; 400 µg) [41], a dsRNA mimetic that activates TLR3 [42] for *Ppargc1a* and *Ppargc1b* rescue in WT and TLR2^−/−^ mice. The polyI:C did not affect *Ppargc1a/b* mRNA levels in HC TLR2^−/−^ mice (Fig. 6C); however, polyI:C in *S. aureus* inoculated TLR2^−/−^ mice showed significant up-regulation of *Ppargc1a* at 6 h PI (4.23-fold vs. HC, *P*<0.05). Because TLR3 activates IRF-3/7 through TRIF, the polyI:C data indicated that IRF3/7 is necessary but not sufficient for *Ppargc1a* induction in this model.

### TLR2-TLR4 signaling

**Figure 7:**
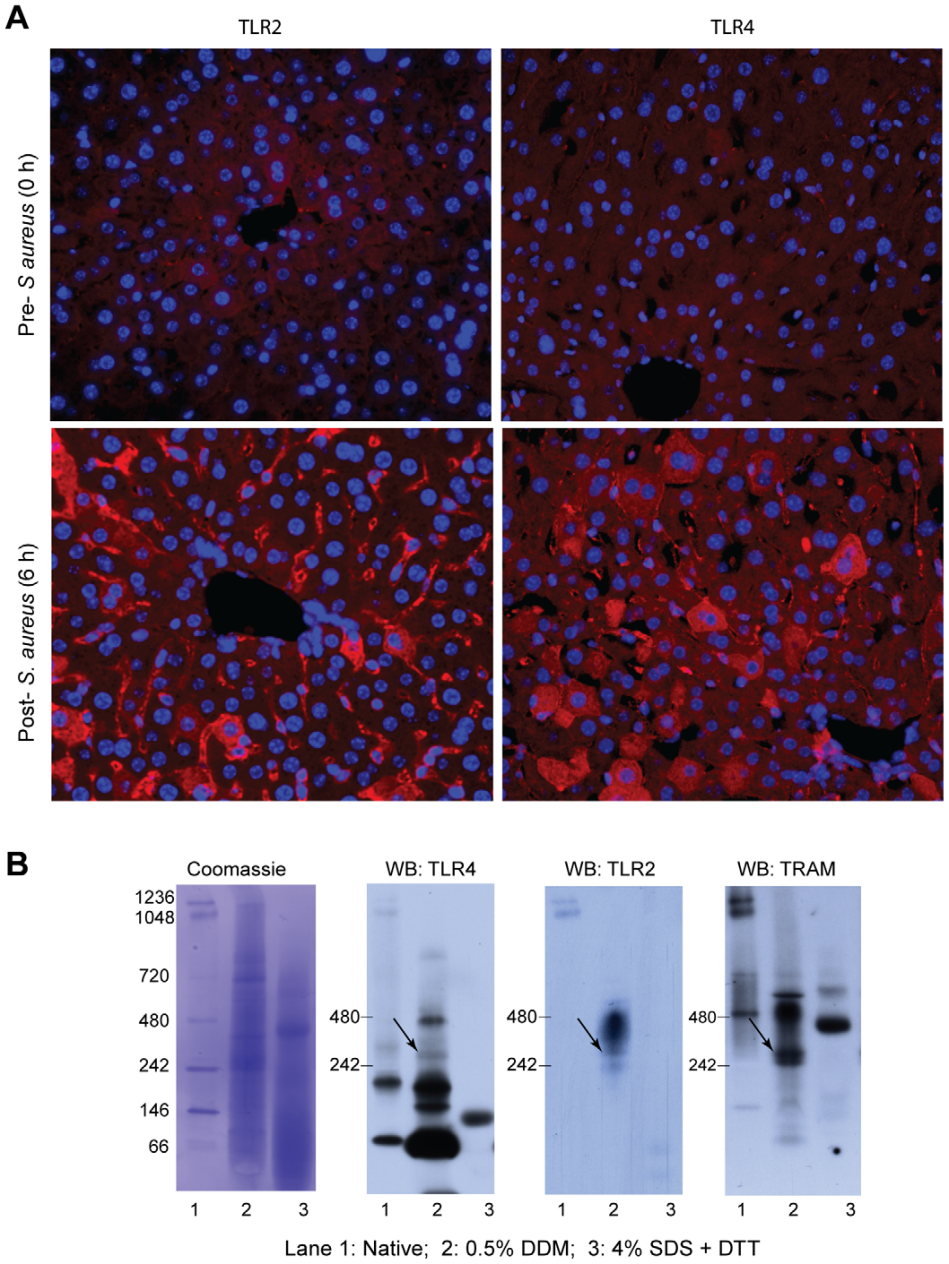
A. TLR2 and TLR4 localization in WT mouse liver by immunofluorescence microscopy. Representative paraffin sections were stained for TLR2 in HC (top left) and 6 h PI (bottom left) and for TLR4 in HC (top right) and 6 h PI (bottom right). TLR staining is red; nuclear staining with DAPI is blue. B. Blue native PAGE on whole liver extracts from WT mice at 6 h after inoculation with *S. aureus*. Each blot shows three lanes: Lane 1, NativeMark molecular weight standard; Lane 2, sample in 0.5% DDM with no DTT or heating; lane 3, sample in 4% SDS with 100 mM DTT, boiled at 95°C for 5 min. At the left, Coomassie staining of entire blot showing molecular markers. Western blots were performed with anti-TLR2, TLR4, or TRAM. A complex near 300 kD was identified by all three primary antibodies (arrows) suggesting a possible interaction among the three proteins.

Since TLR2 ligands act mainly through MyD88, MyD88-independent effects have drawn little further attention since the original studies [7], [43]. Macrophage and dendritic cells stimulated with TLR2 ligands show no ISRE-binding activity or interferon-β (IFN-β) up-regulation or IRF-3 translocation [6], [44], [45], [46]. However, viral particles do activate TRIF and IRF3/7 by TLR2 dimerization with TLR4, leading to TLR2-dependent TLR4 activation and signaling through TRIF and TRAM [9]. TLR-integrin constructs also form TLR2/4 dimers [47], while complementation assays demonstrate cytoplasmic TLR2-TLR4 binding [48]. In macrophages, damage-associated ligands, e.g. biglycan, signal jointly through TLR2 and TLR4 [16], [49]; reviewed in [50]. Using immunohistochemistry, we found that *S. aureus* inoculation simultaneously and widely up-regulates both TLR2 and TLR4 by 6 h in WT mouse liver (Fig. 7A). To explore non-canonical TLR2 and TLR4 interactions in this setting, we compared the native (complexed) and reduced states by Blue native PAGE [51], [52] in pre- and post-inoculation WT liver extracts prepared at 6 h PI in 0.5% *n*-dodecyl β-D-maltoside (DDM) without denaturing (no DTT or heating), or in 4% sodium dodecyl sulfate (SDS) plus 100 mM DTT with denaturing. The membranes were independently blotted for TLR2, TLR4, and TRAM (Fig. 7B). In non-denaturing conditions, each of the three antibodies independently identified the same complex at ∼300 kD.

**Figure 8:**
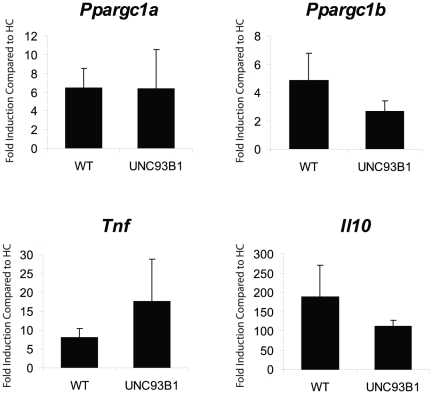
*Ppargc1a*, *Ppargc1b*, *Il10*, and *Tnf* mRNA levels in Unc93b1^−/−^ mice. Hepatic mRNA levels of *Ppargc1a*, *Ppargc1b*, *Il10*, and *Tnf* were measured in healthy controls (HC) and in *S. aureus* sepsis at 6 h PI in WT and Unc93b1^−/−^ mice. There was no significant difference between induction levels in WT and Unc93b1^−/−^ mice for the four genes (n≥3 mice at each point for each strain). Vertical bars are SD.

We also exposed TLR2^−/−^×TLR4^−/−^ mice to *S. aureus*, but in three trials of paired mice, only two survived for 6 h and were moribund, indicating that the inoculation stress was overly severe in the absence of either TLR2 or TLR4. Since a TLR2/4 independent contribution to *Ppargc1a/Ppargc1b* induction had not been excluded, we exposed *Unc93b1*-mutant (3d) mice (deficient in TLR3, 7, 8 and 9 signaling) to *S. aureus* because the unc93b1 protein functions in ER trafficking and mediates translocation of nucleotide-sensing TLRs from endoplasmic reticulum to endolysosomes, allowing for their activation by microbial nucleic acids [53], [54]. This 3d mouse lacks endosome-dependent TLR signaling and its responses signify a role for nucleotide-sensing TLRs in gene activation. We measured *Ppargc1a, Ppargc1b*, *Tnf*, and *Il10* mRNA levels in *Unc93b1* ^−/−^ mice and found that all four genes responded similarly to WT mice (Fig. 8), but there were trends towards more *Tnf* and less *Il10* activation at 6 h PI. Thus, TLR 3 or 7–9 do not regulate *Ppargc1a* and *Ppargc1b* gene expression in *S. aureus* sepsis, suggesting the TLR2/TLR4 balance is specifically involved in the regulation of these genes.

## Discussion

The key new finding is the existence of previously unsuspected NF-kβ-independent transcriptional cross-talk between hepatic TLR2 and TLR4 and the *Ppargc1a* and *Ppargc1b* metabolic co-activator genes. Metabolic dysfunction and organ failure are common and potentially lethal problems in sepsis where pro-survival energy-sensing pathways must be activated in concert with the innate host defenses. Information on the regulation of energy-sensing functions in this setting is limited, but the response is controlled by an integrated transcriptional network that includes NF-kβ [55] and the mitochondrial damage response [56]. Mitochondrial DNA copy number falls in several organs in sepsis, which puts oxidative phosphorylation at risk. The restoration of mitochondrial density is delayed in TLR2 or TLR4 knockout mice relative to WT controls [29].

As crucial co-activators of mitochondrial biogenesis [24], [25], the loss of *Ppargc1a* and *Ppargc1b* function results in a decline in mitochondrial DNA copy number and ultimately in mitochondrial dysfunction [57]. Specifically, in *S. aureus* infection, WT mice up-regulate *Ppargc1a* and *Ppargc1b*, butTLR2^−/−^ mice do not, while TLR4^−/−^ mice display much greater increases in these mRNA levels than do WT mice. Our findings also indicate that these metabolic co-activator genes are regulated by a novel MyD88-independent mechanism.

TLR2 ligands rapidly activate NF-κB, so we first checked for NF-κB regulation of *Ppargc1a* and *Ppargc1b* and found no evidence for involvement of the classical pro-inflammatory NF-κB pathway. TLR2^−/−^ mice surprisingly showed higher and TLR4^−/−^ mice lower early-phase cytokine levels after *S. aureus* compared with WT mice. Moreover, p50^−/−^ and BAY-11-7082-treated mice exhibited *Ppargc1a* up-regulation that was comparable to WT controls, implying that NF-kB activation is not required. Moreover, *Ppargc1a/b* is induced in MAL^−/−^ and MyD88^−/−^ mice after *S. aureus*, even though both lacked NF-κB activation demonstrated by weak *Tnf* expression.

Since *Ppargc1a/b* induction was not impaired in either MAL^−/−^ or MyD88^−/−^ mice, we tested TRAM^−/−^ and TRIF^−/−^ mice and found, like the TLR2^−/−^ mice, that neither strain induced these genes [38]. The main downstream signal of TRAM/TRIF is the phosphorylation of IRF-3 and IRF-7, and our data indicated that nuclear IRF-7 increases in TLR4^−/−^ and decreases in TLR2^−/−^ mice compared with WT mice, reflecting the levels of *Ppargc1* mRNA and the mitochondrial fatty acid oxidation enzyme VLCAD.

The proximal promoter regions of *Ppargc1a/b* in the mouse and human contain multiple partially-conserved ISRE sites, and by ChIP assay the one that spans −289 bp from the *Ppargc1a* transcription start site (TSS) was activated after *S aureus* infection. Given IRF-7 binding to *Ppargc1a*, we exposed IRF-3^−/−^×IRF-7^−/−^ double-knockout mice to *S. aureus* sepsis and saw impaired *Ppargc1a/b* up-regulation, documenting a role for IRF-3/7. Using TLR3 agonist PolyI:C to induce IRF-7 in TLR2^−/−^ mice, we found no increase in basal *Ppargc1a* mRNA levels, but we did rescue the *Ppargc1a* response in sepsis. Thus, other factors are also involved in IRF-7 induction of *Ppargc1a*, e.g. similar to the type I interferon response that follows TLR2 translocation to endolysosomes after ligand engagement [58], though this response is not typical of *S. aureus* sepsis. In any case, *Ppargc1a* induction in mice in response to *S. aureus* infection clearly involves IRF-7, and the TRAM/TRIF→IRF-7→*Ppargc1a/b* pathway represents a broadening of the scope of TLR2 functionality to encompass a rapid metabolic response.

Some intriguing differences in cytokine regulation were also observed in WT, TLR2^−/−^, and TLR4^−/−^ mice, but these were not pursued due to insufficient information on the membrane proteins involved and the known discrepancies in vivo and in vitro in response to live *S. aureus* and to Gram-positive cell wall constituents, e.g. in TLR4^−/−^ mice [10], [11], [12], [13], [14], [15], [16], [29]. Because TLR4 does not bind Gram-positive ligands, a requirement for TLR4 in this in vivo study suggests the possibility that endogenous ligands are involved in the induction of TLR2/TLR4 interactions that are not found in cell systems. For example, fibrin breakdown products and physiological factors absent in cell systems are present in peritonitis models, such as altered intestinal epithelial barrier function, and generate additional DAMPs (e.g. extracellular matrix products) or PAMPs (e.g. LPS translocation).

In this respect, the clot model mimics clinical peritonitis where physical damage and deposition of hemoglobin and fibrin form a bed for infection, and endogenous cell-surface or damage-receptor ligands may contribute to the TLR2/4 interaction. Whatever factors are responsible — microbial or host— TLR4 participates in the defense against *S. aureus*. TLR4^−/−^ mice show decreases in *S. aureus* clearance and increases in mortality similar to TLR2^−/−^ mice [11], [29], and although *Ppargc1a/b* is essential for metabolic gene expression and for mitochondrial biogenesis, these are not the sole survival genes in the intact animal.

**Figure 9:**
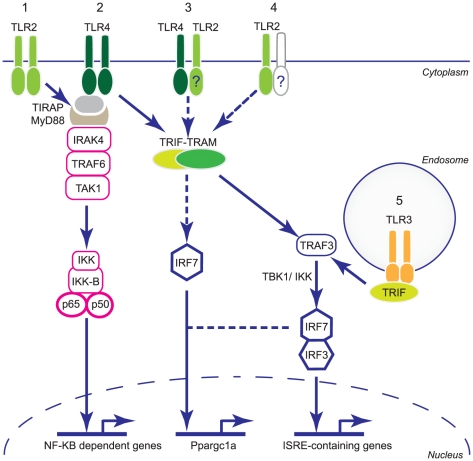
Potential TLR signaling pathways for *Ppargc1* metabolic co-activator gene activation after *S. aureus* infection. Pathway 1 shows the canonical TLR2 MyD88-dependent signaling pathway that activates NF-kB after *S. aureus*. Pathway 2 shows TLR4 MyD88-dependent signaling to NF-kB and MyD88–independent signaling to TRIF/TRAM. Both MyD88 pathways have been excluded as causes of the *Ppargc1a* gene expression. Pathway 3 shows a putative TLR2-TLR4 heterodimer interacting with TRIF/TRAM. Pathway 4 indicates TLR2 in the TLR4 null state, as a homodimer or a heterodimer involving a non-TLR4 partner such as TLR1 or 6, interacting with TRIF/TRAM and unmasking the innate immune regulation of *Ppargc1a* expression. Pathway 5 shows canonical TLR3 endosome signaling also excluded in *Ppargc1* gene regulation after *S. aureus*; however, independent TLR3 activation partially rescues the *Ppargc1* phenotype in mice. TIRAP is Toll/interleukin-1 receptor domain-containing adapter protein (MAL); IRAK4 is Interleukin-1 receptor-associated kinase 4; TRAF3 and TRAF6 are TNF receptor-associated factor 3 and 6; TAK1 is TGF-beta-activated kinase 1 and TBK1 is NF-kappa-B-activating kinase.

The formation of a TLR2-TLR4-TRAM complex may have important implications for the host response to sepsis, but this aspect is preliminary and there is insufficient data to propose a definitive role for it the initiation of the hepatic response to *S. aureus* inoculation. *S. aureus* rapidly up-regulates TLR2 and TLR4 in the mouse liver, and the use of weak non-ionizing detergent and non-reducing conditions allows the detection of a native complex that appears at a molecular weight ∼60 kd higher than the predicted triplex. This implies that one or more other factors, such as post-translational modifications or adapter or chaperone molecules, are involved. The establishment of a functional role for a TLR2-TLR4 -TRAM complex would require cell studies beyond the scope of this in vivo paper, and any such complex could be unique to the liver [2]–[8]. There are multiple potential TLR2 interactions that might explain both the known mitochondrial protective effects of TLR4 and the pronounced up-regulation of the *Ppargc1* metabolic co-activator genes observed here when TLR4 is genetically deleted. Based on our findings, some possibilities for TLR2-TLR4-TRAM interactions leading to IRF7 activation are illustrated in Figure 9. The diagram puts our findings into the context of the well-known TLR2 and TLR4 signaling pathways and outlines testable possibilities for TLR2-dependent MyD88-independent IRF7 activation.

In any case, the TLR2-dependent regulation of *Ppargc1a* and *Ppargc1b* through a MyD88-independent pathway has been established, and this finding not only extends TLR2 signaling to encompass key metabolic genes, but identifies distinct regulation of the NF-kB-dependent pro-inflammatory genes and the hepatic metabolic genes that maintain energy production and initiate mitochondrial biogenesis after *S. aureus* infection. Although the receptor signaling pathways will require detailed molecular and cellular studies, the in vivo biology does suggest novel therapeutic approaches. If *Ppargc1a/b* activation by IRF-7 translates to the clinical setting, it should be possible to establish whether this pathway protects metabolic and organ function during sepsis. *Ppargc1a* and *Ppargc1b* expression increase functional mitochondrial mass [24], [25] and preserved mitochondrial function, e.g. in skeletal muscle, is associated with better outcomes in sepsis [59]. Thus, timed interventions to manipulate IRF-7 may improve cell protection and hasten the resolution of multiple organ failure in patients with sepsis and mitochondrial dysfunction.

## Materials and Methods

### Mouse Studies

The use of mice and the mouse protocols were pre-approved by the Duke University Institutional Animal Care and Use Committee (#A208-10-08). Mice of either gender weighing 20–30 grams were used for the studies. Studies were conducted in wild-type (WT) C57Bl/6J mice obtained from Jackson Laboratories (Bar Harbor, ME) and TLR2^−/−^, TLR4^−/−^, MyD88^−/−^, MAL^−/−^, TRAM^−/−^, and TRIF^−/−^ mice obtained from Shizuo Akira, Japan, and backcrossed >10 generations onto the C57Bl/6J background [10], [60], [61], [62], [63], [64]. TLR2^−/−^×TLR4^−/−^ mice were bred by the Paul Noble laboratory at Duke by crossing TLR2^−/−^ with TLR4^−/−^ mice. The IRF3^−/−^×IRF7^−/−^ mice on the C57Bl/6J background was a kind gift of Dr. Michael Diamond, Washington University St Louis [40]. The p50^−/−^ mice on C57Bl/6J backgrounds were obtained from Jackson. Unc93b1 mice were obtained from the Mutant Mouse Regional Resource Center (Davis, CA) [53].

Mice were anesthetized with intraperitoneal xylazine and ketamine, the abdomen shaved and cleaned with povidone-iodine, and a midline laparotomy was performed. The peritoneum was inoculated with a fibrin clot containing S. aureus and the incision closed in two layers. Mice were resuscitated with 1 ml of subcutaneous 0.9% NaCl. Healthy control (HC) mice of each strain were also used. Mice were humanely killed at 6, 24, 48, or 72 h post-injury (PI) and the livers immediately harvested to isolate mitochondria or snap-frozen and stored at −80°C.

For the clots, Staphylococcus aureus ssp aureus (ATCC) was reconstituted and suspended in bovine fibrin [22]. The bacteria were inoculated sterilely onto agar slants for 18 h and then re-suspended to a concentration of 10^10^ CFU/ml based on optical density at 550 nm. Doses of 10^5^, 10^6^, or 10^7^ CFU were then suspended in 500 µl fibrin clots (500 µl of 10 mg/ml bovine fibrinogen, fraction 1, plus 10 µl of bovine plasma thrombin) (Sigma, St Louis, MO). Pour plates were used to confirm microbial counts. The Limulus Amebocyte Lysate (LAL) assay was performed with a GenScript Chromogenic LAL endotoxin assay kit (GenScript, Piscataway, NJ). Thrombin and fibrinogen were prepared in the standard fashion and tested in duplicate for endotoxin.

### Real-Time RT-PCR

RNA was extracted with TRIzol reagent (Invitrogen, Oslo, Norway) and reverse transcribed with the ImProm-II Reverse Transcription System (Promega, Madison, WI). Mouse-specific primers were designed or purchased from Applied Biosystems (Table 1) and real-time PCR carried out in triplicate, using 18 s primers as internal controls [56]. Real-time PCR output for HC mice of each strain was set to one, and relative quotients obtained at the later time points.

**Table 1:** Antibodies and Primers.

Protein	Host	Company	Catalog #
IRF3	Rabbit	Santa Cruz	9082
p-IRF3	Rabbit	Cell Signaling	4947
IRF7	Mouse	Santa Cruz	74472
IRF7	Mouse	Novus Biologicals	NBP1-04309
LDH	Goat	Santa Cruz	27230
MyD88	Rabbit	Santa Cruz	11356
NF-kB p50	Rabbit	Santa Cruz	7178
NF-kB p65	Rabbit	Santa Cruz	372
TICAM-2 (TRAM)	Rabbit	Santa Cruz	67061
TLR2	Rabbit	Cell Signaling	2229
TLR2	Rabbit	Santa Cruz	10739
TLR2	Rabbit	Rockland	600-401-956
TLR4	Mouse	Imgenex	IMG-5031A
PGC-1α	Rabbit	Cayman	101707
VLCAD	Mouse	MitoSciences	MS707

ChIP Probes	Site	Forward Primer	Reverse Primer
IRF7 on PGC-1β	−467 Bp (from TSS)	CCTCCCTCTCTGGCCTTGCTG	GGGAGGCTTAGAGCCGCTT
IRF7 on PGC-1α	−2395 Bp	AGGGCAGCGTGTCTGTGTTCA	GCTCACACTGAATTGTGGCAGG
IRF7 on PGC-1α	−221 Bp	CAAAGCTGGCTTCAGTCACA	TTGCTGCACAAACTCCTGAC
18S	NR_003278.1	AAACGGCTACCACATCCAAG	CCTCCAATGGATCCTCGTTA

Applied Biosystems	Assay #	Gene Region	Amplicon Length (Bp)
TNF-α	NM_013693.2	mm99999068	Exons 3–4, nt 448 (63)
IL-6	NM_031168.1	mm00446190	Exons 2–3, nt 236 (78)
IL-10	NM_010548.1	mm00439616	Exons 3–4, nt 452 (85)
PGC-1α (*Pargc1a*)	NM_008904.2	mm00447187	Exons 9–10, nt 2072 (82)
PGC-1β (*Ppargc1b*)	NM_133249.2	mm01258518	Exons 9–10, nt 2732 (83)

### Nuclear Isolation

Fresh liver was homogenized in a nuclear buffer (0.32 M sucrose, 3 mM MgCl_2_, 2 mM DTT, 20 mM K-HEPES, pH 7.2, plus 1 µM Na-ascorbate and 1∶100 fresh Sigma Protease Inhibitor Cocktail, Phosphatase Inhibitor Cocktail 1 and Phosphatase Inhibitor Cocktail 2) (Sigma, St Louis, MO). The homogenate was twice-filtered through cheese cloth and centrifuged at 3,800×g for 20 min at 4°C. The supernatant was discarded and the pellet re-suspended in 1.0 ml isolation buffer (2 M sucrose, 1 mM MgCl_2_, 2 mM DTT, 5 mM K-HEPES (pH 7.2)). This suspension was poured over 2.0 ml of isolation buffer and centrifuged at 113,000×g for 1 h at 4°C. The supernatant was decanted and the pellet re-suspended in 150 mM KCl, 5 mM MgCl_2_, 5 mM HEPES buffer at pH 7.2. This suspension was spun at 500×g for 20 min at 4°C, and the pellet was fixed for ChIP analysis or frozen at −80°C in RIPA buffer (150 mM NaCl, 1 mM EDTA, 50 mM Tris-HCl (pH 7.4), 1% Igepal, 0.25% deoxycholate, plus 1∶100 fresh Protease Inhibitor Cocktail, Phosphatase Inhibitor Cocktail 1 and Phosphatase Inhibitor Cocktail 2, and 1 mM PMSF). Nuclear extractions were confirmed by immunoblots for His3 (positive) and LDH (negative) (Santa Cruz Biotechnology, Santa Cruz, CA).

### Western Blots

Whole cell extracts or nuclei were sonicated and standardized for protein using bicinchoninic acid. Proteins were resolved by sodium dodecyl sulfate-PAGE on 4–20% gels and transferred to PDVF membranes. Membranes were probed with affinity-purified primary antibodies (Table 1) and exposed to the appropriate secondary antibody (Santa Cruz). Membranes were developed with ECL (Santa Cruz) and imaged on X-ray film in the mid- dynamic range. Membranes were stained with Coomassie blue as a loading control. The blots were quantified on a BioRad G-710 densitometer.

### Tissue Immunofluorescence

Livers were fixed in 4% paraformaldehyde, dehydrated, paraffin-embedded, and cut into 4–5 micron sections. After antigen retrieval, the slides were stained with primary TLR2 or TLR4 antibody (SC-52735, mouse monoclonal, and SC-10741, rabbit polyclonal, Santa Cruz), a fluorescently-labeled secondary, and counterstained with DAPI. Confocal images were collected in fluorescence mode followed by electronic image merging.

### Chromatin Immunoprecipitation

Nuclear extracts were exposed to 1% formaldehyde for 15 min at 24°C, and the reaction quenched in 0.125 M glycine for 5 min. DNA was sheared with a sonicator into ∼200–800 bp fragments. ChIP was carried out using the ChIP-IT Express Kit (Active Motif, Carlsbad, CA) and the manufacturer's instructions using mouse monoclonal anti-IRF-7 and rabbit polyclonal anti-Pol-II (Santa Cruz Biotechnology). Primers were designed for the ISRE sequence for IRF-7 in the Ppargc1a promoter and the promoter of EF1α (for Pol-II). Conventional PCR was carried out to 40 cycles at 60°C.

### Blue Native PAGE

Snap-frozen liver from WT mice was homogenized in Native PAGE bis-tris buffer (Invitrogen) with either 0.5% DDM or 4% SDS. The lysates were centrifuged at 14,000× g for 20 min, and supernatant protein content measured. DTT was added to a final concentration of 100 mM, and the samples boiled for 5 min at 95°C. The samples were mixed 1∶1 with Blue Native running buffer from the NativePAGE™ Novex® Bis-TrisGel System (Invitrogen), and run on 3–12% bis-tris polyacrylamide gels with NativeMark unstained molecular weight standards (Invitrogen). Gels were transferred to PVDF and washed twice in methanol to remove excess Coomassie blue before immunoblotting.

### Statistics

Grouped data are presented as means ± SD. The n values in the experiments are for the total number of mice of each strain. Each point in the real-time PCR experiments was compared to the healthy control (HC) of its own strain using the Student's t-test. The 6 h between strain points were compared with Student's t-tests with adjustment for multiple comparisons where necessary. The statistical significance levels (P) are provided with the Results.

## Supporting Information

Table S1 **Mouse (Mm)** ***Ppargc1a*** **and human (Hs)** ***PPARGC1A*** **promoter alignment.** ChIP primer sites and the IRF7 consensus sequence for the mouse are indicated. TSS = transcription start site. Note the presence of expanded ISREs in the Hs promoter around the same site.
(DOCX)
