# Supplementary material for: A Toll-Like Receptor 2 Pathway Regulates the Ppargc1a/b Metabolic Co-Activators in Mice with Staphylococcal aureus Sepsis
Source: PLoS One. 2011 Sep 26;6(9):e25249. doi: 10.1371/journal.pone.0025249 (PMC3180377; doi:10.1371/journal.pone.0025249)
Supplement: Table S1 — Mouse (Mm) Ppargc1a and human (Hs) PPARGC1A promoter alignment. ChIP primer sites and the IRF7 consensus sequence for the mouse are indicated. TSS = transcription start site. Note the presence of expanded ISREs in the Hs promoter around the same site. (DOCX) [file pone.0025249.s001.docx]

**Sweeney et al Supplemental Table 1**
